# Anti-inflammatory activities of flavonoid derivates

**DOI:** 10.5599/admet.1918

**Published:** 2023-07-26

**Authors:** Ysrafil Ysrafil, Zulfiayu Sapiun, Nangsih Sulastri Slamet, Fihrina Mohamad, Hartati Hartati, Sukmawati A Damiti, Francisca Diana Alexandra, Sudarman Rahman, Sri Masyeni, Harapan Harapan, Sukamto S. Mamada, Talha Bin Emran, Firzan Nainu

**Affiliations:** 1 Department of Pharmacotherapy, Faculty of Medicine, Universitas Palangka Raya, Palangka Raya 73111, Indonesia; 2 Department of Pharmacy, Politeknik Kesehatan Kementerian Kesehatan Gorontalo, Gorontalo 96135, Indonesia; 3 Department of Midwivery, Politeknik Kesehatan Kementerian Kesehatan Palangka Raya 73111, Palangka Raya, Indonesia; 4 Faculty of mathematics and natural sciences, Universitas Palangka Raya, Palangka Raya 73111, Indonesia; 5 Department of Internal Medicine, Faculty of Medicine and Health Sciences, Universitas Warmadewa, Denpasar, Bali 80235, Indonesia; 6 Department of Internal Medicine, Sanjiwani Hospital, Denpasar, Bali 80235, Indonesia; 7 Medical Research Unit, School of Medicine, Universitas Syiah Kuala, Banda Aceh 23111, Indonesia; 8 Tropical Disease Centre, School of Medicine, Universitas Syiah Kuala, Banda Aceh 23111, Indonesia; 9 Department of Microbiology, School of Medicine, Universitas Syiah Kuala, Banda Aceh 23111, Indonesia; 10 Department of Pharmacy, Faculty of Pharmacy, Hasanuddin University, Makassar 90245, Indonesia; 11 Department of Pathology and Laboratory Medicine, Warren Alpert Medical School & Legorreta Cancer Center, Brown University, Providence, RI 02912, USA; 12 Department of Pharmacy, Faculty of Allied Health Sciences, Daffodil International University, Dhaka 1207, Bangladesh

**Keywords:** inflammation treatment, bioactive compounds, plants-base drug, nanotechnology

## Abstract

**Background and purpose:**

Flavonoids are a group of phytochemicals found abundantly in various plants. Scientific evidence has revealed that flavonoids display potential biological activities, including their ability to alleviate inflammation. This activity is closely related to their action in blocking the inflammatory cascade and inhibiting the production of pro-inflammatory factors. However, as flavonoids typically have poor bioavailability and pharmacokinetic profile, it is quite challenging to establish these compounds as a drug. Nevertheless, progressive advancements in drug delivery systems, particularly in nanotechnology, have shown promising approaches to overcome such challenges.

**Review approach:**

This narrative review provides an overview of scientific knowledge about the mechanism of action of flavonoids in the mitigation of inflammatory reaction prior to delivering a comprehensive discussion about the opportunity of the nanotechnology-based delivery system in the preparation of the flavonoid-based drug.

**Key results:**

Various studies conducted in silico, in vitro, in vivo, and clinical trials have deciphered that the anti-inflammatory activities of flavonoids are closely linked to their ability to modulate various biochemical mediators, enzymes, and signalling pathways involved in the inflammatory processes. This compound could be encapsulated in nanotechnology platforms to increase the solubility, bioavailability, and pharmacological activity of flavonoids as well as reduce the toxic effects of these compounds.

**Conclusion:**

In Summary, we conclude that flavonoids and their derivates have given promising results in their development as new anti-inflammatory drug candidates, especially if they formulate in nanoparticles.

## Introduction

Inflammation is a complex and important protective response of the body to a stimulus in the form of stimulation by microorganisms, physical injuries, chemicals, allergic reactions or the presence of endogenous signals due to cell damage. In a physiological state, inflammation aims to eradicate inflammatory agents as well as repair the injured tissue and wounds [1-3]. In the aspect of pathomechanism, inflammation involves the body's immune system response, both natural and adaptive, although in most cases, the response is mainly initiated by the natural immune system. Inflammation is initially stimulated by the introduction of highly conserved pathogen- or damaged- associated molecular patterns (PAMPs and DAMPs) by pattern recognition receptors (PRRs) of the immune cells [4-6]. This will trigger the subsequent activation of the inflammatory cells such as neutrophils, eosinophils, mononuclear phagocytes, and macrophages, leading to the excessive production of nitric oxide (NO), prostaglandin E2 (PGE2), C-reactive protein (CRP), chemokines and proinflammatory cytokines such as interleukins (IL)-1β, IL-6, and tumour necrosis factor (TNF)-α.

The production of proinflammatory cytokines generally occurs due to the induction of nuclear factor-kappa B (NF-κB), a transcription factor that plays a key role in the regulation of inflammation. In addition to the release of several molecules above, the inflammatory process is also accompanied by the release of a number of bioactive lipid mediators such as thromboxane, prostaglandins and prostacyclins on the initiative of the phospholipid conversion cascade by phospholipase and cyclooxygenase enzymes. The release of cytokines, chemokines, and mediators and these inflammatory molecules then triggers the appearance of inflammatory signs such as calor, dolor, rubor, tumour and *functio laesa*, and if it continues, it can cause a worse impact [3,7].

However, the protective function of inflammation can shift to the detrimental effects caused by the uncontrolled and excessive inflammatory response. In this condition, inflammation may disturb the homeostasis of the body's physiological processes and eventually develop into an inflammatory disorder [1-3]. Several studies have demonstrated that inflammation is associated with developing and worsening various non-communicable disease-associated inflammation disorders, such as autoimmune diseases, neurodegenerative diseases, cancer, diabetes mellitus, and cardiovascular diseases [1,8]. These findings support the development of anti-inflammatory drugs to prevent the progression of the diseases.

Advances in the last few decades have revealed many facts about the benefits of various plant metabolites against inflammation. Curcumin, resveratrol, and capsaicin have been previously reported to have the ability to decrease the production of the proinflammatory cytokines IL-1β, IL-6, and TNF-α, inhibit cyclooxygenase-2 (COX-2) and the inflammatory pathways (e.g., NF-κB, mitogen-activated protein kinase (MAPK), and activator protein 1 (AP-1) [9,10].

Of several plant metabolites with potential anti-inflammatory action, flavonoids have attracted much interest. Several studies have reported the activity of flavonoids in interfering with inflammatory signalling pathways and key enzymes, leading to the inhibition of the release of pro-inflammatory proteins and mediators [8,11]. However, the current problem is that these compounds have poor bioavailability and pharmacokinetic profiles. Advances in nanotechnology have brought promising approaches in improving the bioavailability and increasing the effectiveness of these compounds as anti-inflammatory drug candidates [12,13].

In our review, we summarize the latest scientific findings regarding the benefits of flavonoids, pharmacokinetics, and their development based on nanomedicine, as this technology has become a solution in the delivery and improvement of drug effectiveness, including drugs used to impede inflammatory conditions.

## Mechanisms of inflammation

As a fundamental biological process, inflammation is the protective response to tackle invading pathogens, antigens or damaged cells or tissues. It provides a defence mechanism to eliminate harmful substances and perform tissue repair [14,15]. At the molecular and cellular level, inflammation is characterized by five major signs: redness, heat, pain, swelling and functional loss. These changes can be triggered by two main causes, including infectious and non-infectious-related causes. The presence of these causes in the body will be recognized by receptors in the body that are subsequently followed by the activation of the inflammatory cascade, the release of the markers, and the recruitment of the inflammatory cells [16].

### The recognition of PAMPs and DAMPs by PRRs

Tissue injury, cell-infected pathogens, microbial invasion, or death cells associated with necroptosis or pyroptosis will release PAMPs or DAMPs. PAMPs are unique structures in microbes or pathogens (*e.g.*, LPS, β-glucan, flagellin, spike, DNA, RNA) that can be recognized by immune cells and trigger their activation [17,18]. Meanwhile, DAMPs are endogenous molecules that are hidden from recognition by the immune system and will only be released when cell damage occurs as a signal to activate innate immune cells Several DAMPs released by damaged cells include high mobility group box 1 (HMGB1), histones, ATP, heat shock proteins, fibrinogen, versican, uric acid, and mitochondrial components such as N-formylated peptides, C-phosphate-G (CpG), DNA repeats, and mitochondrial DNA (mtDNA) [19].

Both PAMPs and DAMPs will be recognized by PRRs presented on innate immune cells such as neutrophils, monocytes, dendritic cells, macrophages, and other inflammatory cells [18,20]. Neutrophils are the first leukocytes migrating to the injured or infected tissue to kill pathogens through the phagocytic mechanism and granule secretion. This process involves the release of reactive oxygen species (ROS), hydrolytic enzymes, antimicrobial peptides, chemokines/cytokines, lipid mediators, as well as neutrophil extracellular traps (NETs) to initiate inflammation and monocyte activation. Upon this process, monocytes and macrophages will also migrate to the site of inflammation within a few hours to further promote inflammation as well as be involved in tissue repair [17,20].

Innate immune cells have various PRRs that recognize DAMPs and PAMPs, trigger phagocytic processes, and mediate inflammation. There are 4 classes of PRRs, including Toll-like receptors (TLRs), NOD-like receptors (NLRs), C-type lectin receptors (CLRs), and RIG-I-like receptors (RLRs) [21,22]. TLRs are a highly conserved subclass of PRRs that are localized in transmembrane (TLR1, 2, 4, 5, 6, and 10) and in intracellular (TLR3, 7, 8, and 9). They can recognize various DAMPs and PAMPs to trigger further activation of the MyD88 signalling pathway or TRIF-dependent signalling pathway to induce the production of proinflammatory cytokines and interferon-gamma. NLRs and RLRs have sensors that are located in the cytoplasm. However, they play different functions. RLRs are generally more specific for recognizing genome viruses (ssRNA and dsRNA) and trigger the production of IFNs and inflammatory cytokines. Meanwhile, NLRs tend to recognize PAMPs associated with bacteria (*e.g.*, peptidoglycans, gamma-d-glutamyl-meso-diaminopimelic acid, and muramyl dipeptide) and some DAMPs (*e.g.*, uric acid, histones, biglycan, hyaluronan). Recognition by these NLRs triggers the activation of the NF-kB signalling pathway [19,23].

Furthermore, CLRs are unique PRRs that are located on the transmembrane and are characterized by the presence of a carbohydrate-binding domain in their receptors. They can recognize several pathogenic molecules (*e.g.*, carbohydrates presented on bacteria, fungi, and viruses) as well as damaged cells (*e.g.* SAP130, F-actin). This recognition will trigger the activation of the NF-kB signalling pathway either through TLR modulation or directly through spleen tyrosine kinase (SYK) and RAF1 pathways. The interaction between CLRs and SYK also triggers the activation of MAP kinase [19,23].

### Lipid-based signaling: arachidonic acid-derived eicosanoids

Recognition of DAMPs and PAMPs by TLRs leads to the rapid phosphorylation of MAPK. The presence of MAPK will lead to the activation of cytosolic phospholipase A2 (cPLA), an enzyme that can cleave membrane phospholipids to produce arachidonic acid (AA) as the primary eicosanoid precursor [24,25]. Cytoplasmic AA can further be metabolized by COX, lipoxygenases (LOX), or cytochrome enzymes (CYP) to produce oxygenated derivatives of eicosanoids including prostaglandins (PGs), thromboxanes (TXs), leukotrienes (LTs), lipoxins (LXs), hydroxyeicosatetraenoic acid (HETEs), epoxyeicosatrienoic acids (EETs), and eicosatetraenoic acids (ETEs). In addition to using arachidonic acid, eicosanoids can also be synthesized from other polyunsaturated fatty acids (PUFAs) such as di-homo-γ-linolenic acid (DGLA), α-linolenic acid (ALA), docosahexaenoic acid (DHA), or eicosapentaenoic acid (EPA). These eicosanoids can increase nerve pain signals and vasodilation in tissues that cause pain and swelling in the injured or invaded area [25-29]. COX is a highly conserved enzyme that plays an important role in the inflammatory process, making it a popular target for therapy like non-steroidal inflammatory drugs (NSAIDs). They exist in two isoforms, namely COX-1 and 2, and are responsible for the conversion of AA to various prostaglandins and thromboxanes [25,27]. Furthermore, LOX (such as 5-LOX, 8-LOX, 12-LOX, and 15-LOX) is an AA-metabolizing enzyme that works by inserting molecular oxygen on its substrate to produce hydroperoxy eicosatetraenoic acids (HPETE). HPETE is an intermediate product that can be further converted to various eicosanoids, including HETEs, LXs, and hepoxilins. Meanwhile, CYP is associated with the metabolism of AA into HETEs and EET on the regulation of inflammation in certain organs such as the heart, blood vessels, and several other organs. However, despite this, drug development through this route is in lack of interest [29]. It is noteworthy that in addition to these afore mentioned enzymes, drug development is also carried out by targeting another enzyme called cytosolic phospholipase A2.

### Release of proinflammatory chemokines and cytokines

Recognition and binding of pathogen and damaged cell molecules by PRRs induce innate immune cells to release small signalling molecules, namely chemokines and cytokines, as a cascade of protective responses against invaded pathogens and repair of tissue injury. Cytokines are small proteins secreted by immune cells to facilitate interaction and communication between cells. These proteins exist in several terms based on their origin, including lymphokine (secreted by lymphocytes) and monokine (secreted by monocytes), interleukins (secreted by certain lymphocytes but acting on other lymphocytes) as well as their activity, *e.g.* chemokines (cytokines with chemotactic activities) [30,31]. Cytokines play an important role in inflammation both as inflammatory stimulants (proinflammatory cytokines) such as IL-1β, IL-6, and TNF-α and those inhibitions of inflammatory (anti-inflammatory cytokines), *e.g.*, IL-10, IL-1RA, IL-4, IL-11, and IL-13. Proinflammatory cytokines are highly secreted by activated and polarized macrophages for the upregulation of inflammatory reactions and mediate remote adaptive immunity during tissue or cell injury, invasion of the pathogen, and inflammation. Some evidence reported that IL-1β can also be secreted by monocytes, fibroblasts, and endothelial cells during the stimulation of PRRs. Expressed IL-1β will be further activated by IL-1-converting enzyme (ICE) or caspase-1 and followed by forming a complex with their receptor. This situation causes signal transduction leading to the activation of MAPK and NF-κβ to produce proinflammatory cytokines. Furthermore, similar to IL-1β, TNF-α is also secreted by other cells, including monocytes, T cells, mast cells, natural killer (NK) cells, keratinocytes, fibroblasts, and neurons. Their proinflammatory effect predominantly appears after their binding to tumour necrosis factor receptor 1 (TNFR1) and further facilitates activation of the c-Jun N-terminal Kinase (JNK), NF-κB, and MAPK signalling pathways. Meanwhile, IL-6, which is also expressed by neutrophils, fibroblasts, both T and B cells, endothelial cells, keratinocytes, hepatocytes, and bone marrow cells, first binds to IL-6R to mediate inflammation through activation of signal transduction via the gp130 proteins and lead activation of the JAK/STAT signalling pathway [32].

The other highlighted cytokine in inflammation is pro-inflammatory chemokine. Chemokines, also known as chemotactic cytokines, are proteins of the cytokine family that play an important role in the chemoattraction and migration of leukocytes to various sites of tissue injury. Inflammatory chemokines will be secreted when there is an inflammatory stimulus (both pathogens or damaged cells) to mediate further the innate and adaptive immune response [30,33]. Chemokines induce the expression of integrins such as 2-integrin into leukocytes to facilitate diapedesis of these cells to the site of injury. Cytokine signalling can be transduced by binding to the seven-transmembrane G protein-coupled receptor (GPCR) found throughout the body [32,33].

### Involvement of MAPK and NF-κβ signalling pathways

In response to the introduction of PRRs to PAMPs or DAMPs activating the innate immune cells like macrophages, a series of intracellular signals are also activated to produce inflammatory mediators such as IL-1β and TNFα. They will trigger a signalling cascade involving the adapter protein MyD88 to lead to activation of the host MAPK pathway, which is further followed by activation of transcription factor NF-κB. This, in turn, induces the production of proinflammatory cytokines [34-36]. Under normal conditions, the presence of IκB proteins in the cytoplasm could inhibit NF-κB. The presence of a stimulus in PRRs triggers signal transduction leading to the formation and activation of IκB kinase (IKK) that is composed of IKKα and IKKβ, as well as IKKγ as the regulatory subunit. IκB will be further phosphorylated to lead activation of NF-κβ. Taken together, both signalling pathways play an important role in the inflammatory processes because they are the suppliers of proinflammatory cytokines and thus serve as targets for drug action [15,21,36].

## Flavonoid and its derivates

**Figure 1.**
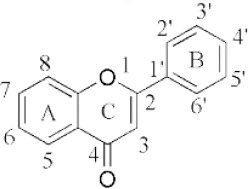
The basic structure of flavonoids.

Flavonoids are a class of compounds with a low molecular weight based on the 2-phenyl-chromone nucleus (Figure 1).

The basic structure of flavonoids consists of aglycones, but their presence in nature is usually bound to glycosides and methylated derivatives. They are produced via the shikimic acid pathway using acetic acid/phenylalanine derivatives as the precursor. Flavonoids are traditionally classed by oxidation degree, ring C annularity, and ring B connectivity [1,37]. They comprise 6 subclasses, as presented in Table 2 [38,39]. Among higher plant families and genera, the flavones and flavonols are the most commonly highly conserved encountered and marked by the presence of the 2-phenylchromen-4-one (2-phenyl-1-benzopyran-4-one) structures in their backbone [40].

Flavones are distinct from other flavonoids because their skeleton contains a double bond between C2 and C3. Also, there is no substitution at their C3 position, while flavonols possess hydroxyl substitution at that position. Further, flavones are oxidized at the C4 position [41,42]. Flavanones and flavanols have saturated bonds between C2 and C3 and frequently coexist in plants with flavones and flavonols [37]. Flavanones have a flavan nucleus formed by two aromatic rings (A and B) linked by a dihydropyrone ring (C). The presence of a saturated C2–C3 bond, a chiral carbon atom at the C2 position, and no substitution at the C3 position of the C ring distinguish flavanones from the other two classes of flavonoids, *i.e.*, flavones and flavanols [43]. Flavanols (flavan-3-ols) are heterocyclic compounds that contain a saturated heterocyclic ring, a single bond between C2 and C3, and a hydroxyl group at the C3 position. Unlike other flavonoids, flavanols are only found in food as aglycones (the glycosylated state is excluded). Additionally, they are found in monomeric form as catechins and epicatechins, as well as in polymeric form as tannins [44]. Isoflavones are secondary metabolites formed by rhizobial bacteria and leguminous plant defence responses [45]. Isoflavones, like daidzein, include 3-phenylchromones. Chalcones are ring C-opening isomers of dihydroflavones that are responsible for plant colour. Anthocyanidins are a category of significant chromene pigments that exist as ions. Other flavonoids without the C6—C3—C6 structure include biflavones, furan chromones, and xanthones. Glycosides are the most common extant flavonoid form. The structure of aglycones dictates preferred glycosylation sites [37].

## Pharmacokinetics and bioavailability

### Bioavailability

To predict and explain the effect of flavonoids at their site of action, knowledge of the pharmacokinetics, form, and concentration of these compounds in plasma must be known. Animal and human studies have found that these compounds have poor oral bioavailability, which might be attributed to the loss of the compounds during the absorption and metabolism phases. In addition, these compounds also tend to have poor water solubility, low permeability, as well as poor stability profile [13,61]. Therefore, various studies have been carried out to engineer their structure to increase their water solubility and bioavailability and thereby increase their activity as a drug candidate as have been demonstrated in the treatment of several diseases [13,46,62].

### Absorption

Absorption of flavonoids in the intestine can occur through several mechanisms, namely active transport, passive diffusion, or both. Some flavonoids, such as quercetin, are absorbed completely in the small intestine by involving sodium-dependent glucose transporters 1 (SGLT1). These transporters are located in the apical membrane of intestinal epithelial cells that utilize Na+/K+-ATPase pumps to transport this flavonoid. In addition, the absorption of this compound is also carried out by glucose transporter 2 (GLUT2) located on the basolateral membrane of the intestine. However, the exact mechanism used by GLUT2 in transporting flavonoids is still unclear [63-65]. Further, quercetin is hydrolysed by the intracellular enzymes called lactase-phloridzin hydrolase (LPH) and cytosolic β-glucosidase (CBG) in the aglycone form so that it is easily absorbed through the small intestine. In addition to quercetin, these enzymes can work on other flavonoid compounds. However, the effectiveness of these enzymes depends on the glycosides present in the flavonoid structure [41,64,66,67]. It should be noted that each compound grouped as flavonoids have different maximum absorption times. A study that was conducted in rats revealed that the absorption of some flavonoid derivatives such as apigenin, luteolin, and glucosides generally occurred rapidly with a maximum absorption time (*t*_max_) of about 1 hour for maximum plasma concentration (*C*_max_) of 1 to 100 μmol/L and it will change depends on co-consumed food [41,68].

### Distribution

In circulation, flavonoids can bind to several blood proteins [69]. Gecibesler & Aydin [70] demonstrated that flavonoids could bind strongly to human serum albumin (HSA). HSA is an essential protein in blood plasma found abundantly in the body [71]. This soluble protein can bind and transport various metabolites and organic compounds such as flavonoids, unesterified fatty acids, hormones, and metal ions to various tissues throughout the body [72,73].

Research by Boer *et al.* [74] revealed that flavonoid compounds, in particular quercetin and its derivatives, are widely distributed in rat tissues with the highest levels in the lungs, testes, and kidneys, while the brain, white fat, and spleen showed the lowest levels. This group also reported that this compound was deposited in several other organs in the rat, including the thymus, heart, liver, brown fat, bone, and muscle. Meanwhile, in pigs, the deposition of quercetin was more abundant in the liver and kidney tissue after a quercetin diet of 500 mg/kg with a lesser extent in the brain, heart, and spleen .

A similar finding was reported by Beiger *et al.* [75] demonstrating that the distribution of quercetin and some of its derivatives was found to be highest deposited in the liver, kidney, jejunum, lung, and muscle (longissimus dorsi) after a single dietary quercetin dose (25 mg/kg) in pigs. Furthermore, quercetin deposition was more distributed when administered at a dose of 50 mg/kg per day for four weeks, including colon (higher), kidney, jejunum, liver, lymph node, mesentery, lung, white adipose tissue, muscle (diaphragm), muscle (longissimus dorsi), and brain (as the smaller deposition).

### Metabolism

After ingestion, approximately 10 % of flavonoid glycosides are absorbed in the upper gastrointestinal tract, while the remaining 90 % pass through the small intestine to reach the large intestine as unmetabolized and unabsorbed flavonoids. The unabsorbed flavonoids undergo a further enzymatic transformation in the small intestine, such as oxidation, reduction, and decarburization, as the preparatory steps before entering the large intestine. Once in the large intestine, they are hydrolysed and cleaved to remove glycosides and produce flavonoid aglycones, which are further metabolized to ring fission products. This process is carried out by the involvement of the lactase-phlorizin hydrolase (LPH) and colonic enzymes produced by the intestinal microbiota [41,76-78].

Flavonoids then extensively undergo two metabolic phases, namely phases 1 and 2, which occur in enterocytes and hepatocytes [79,80]. In the first phase, which takes place in hepatocytes, flavonoids will be oxidized by the cytochrome P450 (CYP450) enzyme to produce a minor contribution to flavonoid clearance. Different isoforms of the CYP450 enzyme play the metabolic process of several flavonoid-derived compounds in this phase. Isoform CYP2C9 is the most efficient enzyme for the metabolism of galangin followed by CYP1A3 and CYP1A1. Meanwhile, most of the oxidation of galangin is determined by the action of CYP1A2 and followed by CYP2C9 and CYP1A1 [81]. Another investigation reported that apigenin was metabolized by CYP1A1, CYP2B, and CYP2E to form three monohydroxylated derivatives [82].

In contrast to the first phase, phase II metabolism occurs in enterohepatic and enteric and is predominant in the direction of flavonoid disposition. In this stage, flavonoids undergo two main processes, namely glucuronidation and sulfation. While the former is mediated by UDP glucuronosyltransferases (e.g., UGT1A1, UGT1A8, and UGT1A9), the latter is catalysed by sulfotransferases (*e.g.*, SULT1A1 and SULT1E1). Methylation can also occur in this phase. The process is mediated by a group of enzymes known as methyltransferases mostly found in many tissues, including the liver and intestines. The most common methylation reaction associated with flavonoid metabolism is O-methylation which occurs in the liver and is catalysed by catechol-O-methyltransferase (COMT) [41,64,69,79,83,84]. The products of these metabolic processes will then be circulated and deposited in various body components to carry out their actions, including their anti-inflammatory activities, as elaborated in the section “Flavonoid derivates as anti-inflammatory agents and their mechanisms of action”.

### Excretion

Flavonoids are mainly excreted through the urine. A study reported that about 60-80 % of the metabolites of anthocyanins are excreted in the urine [85,86]. The excretion pathway of flavonoids depends on the conjugate form and the site of elimination. Glucuronide formed in the intestine tends to enter the systemic circulation directly and is excreted in the urine, while the products formed in the liver are mainly eliminated through the bile. This indicates that glucuronidation in enterocytes Favors the loss of flavonoids from the enterohepatic circulation (EHC) into the systemic circulation.

EHC-undergoing flavonoid compounds may also be further sulphated in the intestine or hepatocytes, leading to urinary excretion (without bypassing biliary excretion). Another study in isolated human faecal colonic bacteria found the presence of flavonoids such as quercetin, indicating the importance of the faecal route in facilitating the excretion of these compounds [76,86,87]. To sum up, the biological activities of flavonoids are diminished by the fact that these compounds have low aqueous solubility and poor bioavailability, as depicted in their pharmacokinetic profile described above. This leads to the development of various strategies to increase their solubility and oral bioavailability, including the development of nanoparticle-based delivery systems, which will be discussed in the section “Application of nanomedicine as delivery system of flavonoid derivates”.

## Flavonoid derivates as anti-inflammatory agents and their mechanisms of action

**Figure 3.**
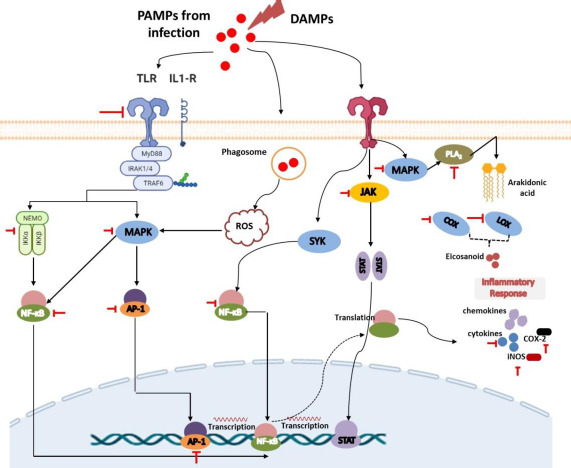
Mechanism of action of flavonoids as an anti-inflammatory agent.

Various studies of in silico, in vitro, in vivo, and clinical trials have demonstrated that flavonoids have potential activities in mitigating inflammation [11,88]. They can modulate various key points in regulating inflammation in the early and late stages. As displayed in Table 1, these compounds consist of several subclasses, and they can exert their anti-inflammatory properties via various mechanisms, including inhibition of the various inflammatory signaling pathways such as NF-κB [88], MAPK [89], blockage of pro-inﬂammatory enzymes (COX-1, COX-2, and 5-LOX) [11], inhibition of proinflammatory cytokines release (TNFα, IL-1β, and IL-6), and suppression of other inflammatory proteins [62]. The summary of the putative anti-inflammatory mechanisms of flavonoids is presented in Table 2 and Figure 3.

### Flavonoid inhibit eicosanoids metabolism and function

Prostaglandins and leukotrienes are potent lipid mediators derived from phospholipase-released arachidonic acid that play a critical role in inflammation [160]. They are produced from locally damaged cell membranes with the help of several enzymes such as phospholipase A2 (PLA2), cyclooxygenase, and lipoxygenases as previously explained (in section Lipid-based signaling: arachidonic acid-derived eicosanoids). Several approved drugs such as corticosteroid-including drugs (such as methylpredisolone *etc*.) and nonsteroidal anti-inflammatory drugs (NSAIDs; *e.g.*, mefenamic acid, indomethacin, ibuprofen) have targeted these enzymes for the treatment of several inflammation-related diseases such as pain, injury, asthma, allergies, and inflammation. Elevated COX-2 levels have indeed shown adverse effects on inflamematory conditions and even on some other inflammation-related diseases such as tumor development, so their suppression could significantly suppress worsening prognosis [161].

Different studies demonstrated that flavonoid-derived compounds can inhibit enzymes, including phospholipase A2, cyclooxygenase, and lipoxygenases, improving inflammatory disorders [52,160,162,163]. Several pieces of evidence have proposed their effect to block the pathway and mediate improvement in an intestinal inflammatory response. A study by López-Posadas [162] reported that flavonoids could inhibit the formation of prostaglandins from the AA pathway by suppressing epithelial COX-2 expression during inflammation in Intestinal epithelial cells. This finding is supported by another study reported by Serra and colleagues that advocated C3G to provide strong inhibitory activity against COX-2 and lead downregulation of several inflammatory mediators in the colonic carcinoma cell [164].

Apart from intestinal inflammation, several flavonoid derivative compounds have also been reported to be effective in inhibiting eicosanoid production in other inflammatory cases. A study by Husain *et al.* suggested that epigallocatechin-3-gallate (EGCG) can downregulate COX-2 in human prostate carcinoma LNCaP and PC-3 cells in a dose-dependent fashion [165]. Similar findings demonstrated the efficacy of EGCG could downmodulation of COX-2 were further lead reduction of PGE2 levels [166,167]. In another finding, giving EGCG results in the inhibition of NF-κB that in order leads to the inhibition of COX-2 promoter activity [168]. Several other flavonoid derivative compounds, such as luteolin, kaempferol, hesperetin, and naringin, have been shown to have inhibitory effects on this COX-2 enzyme [161,169]. Topical pre-treatment of kaempferol could attenuate UVB-induced COX-2 expression in mouse skin. This effect is hypothesized to arise through the modulation of Src kinase activity in the mouse skin [161]. Another promotion study demonstrated by He and colleagues [170], isolated and elucidated flavonoid compounds from *Hosta plantagine* and found twelve 12 different ones that showed significant inhibitory effects to both enzyme isoforms from 53.00 to 80.55 % (in COX-1) and 52.19 to 66.29 % (to COX-2) in concentration 50 μM.

The increase in eicosanoids is also mediated by the COX-1 isoform, especially in some cases of neuroinflammation. Elevated COX-1 levels in microglia may explain PGE2 levels in the cerebrospinal fluid of Creutzfeldt-Jacob disease (CJD) patients associated with shorter survival [171]. Regarding that, it has been reported that quercetin and tomentodiplacone-O could strongly inhibit COX-1 [11]. Similar to this finding, Kaempferol has been advocated to have an inhibitory effect on COX-1 in vitro. In addition to the cyclooxygenase pathway, several researchers have reported that this compound can inhibit the expression of lipooxygenase (LOX) enzymes which play an important role in the metabolism of arachidonic acid into leukotrienes, which is involved in various inflammatory-related diseases including asthmatics, rheumatoid arthritis, psoriasis, inflammatory bowel disease *etc*. [51,172]. Administration of several other flavonoid derivative compounds, such as quercetin and luteolin has also been shown to have a strong inhibitory effect on these enzymes to lead to improvement in symptoms of inflammation-related leukotrienes expression [169].

The effectiveness of flavonoids in inhibiting the formation of eicosanoids in the AA-related pathway has also been proposed through the PLA2 enzyme inhibition mechanism. This enzyme plays an important role in the process of converting membrane phospholipids into product intermediates, namely arachidonic acid, before being further converted into lipid mediators. Many recent studies have reported the activity of various flavonoid derivative compounds having good activity in inhibiting this enzyme and treating inflammation [173,174]. A study conducted by Enechi and reported colleagues [175] that flavonoid-rich extract from *Peltophorum pterocarpum* stem-bark provide a strong inhibitory effect in PLA2 and generate downmodulate of the inflamematory symptoms of paw oedema induced by egg albumin, in comparison with prednisolone as well approve anti-inflammatory drugs that act in PLA2. In accordance, several other studies have also demonstrated compounds such as quercetin, kaempferol, and galangin showing promising inhibitory activity against PLA2 [173,174,176].

### Flavonoid inhibit MAPK and NF-κβ signalling pathways

Various evidence has suggested the crucial role of Nuclear Factor Kappa Beta (NF-κβ) and Mitogen-activated protein kinases (MAPKs) as two main signaling pathways that are interrelated in underlying inflamemation [177]. NF-κβ accumulation has been implicated with increased transcription and expression of various inflammatory mediators. Meanwhile, MAPKs, consisting of p38 kinase, ERK, and c-Jun NH2-terminal kinase (JNK), have been advocated to regulate the transcription of COX-2, iNOS, and inflammatory cytokines in inflammation. Therefore, increasing evidence has studied and targeted these signaling pathways in the development of anti-inflammatory drugs [177-179]. A study conducted by Hour and colleagues [177] reported that dihydromyricetin, a flavonoid isolated from *Ampelopsis grossedentata* has been proposed to have good and promising inflamematory potential by inhibiting the phosphorylation of NF-κB and IκBα as well as p38 and JNK. This suppression simultaneously leads to a downmodulation effect on various proinflammatory cytokines such as TNF-α, IL-1β, and interleukin-6, as well as increasing levels of IL-10 as an anti-inflammatory cytokine. Furthermore, inhibition of these two signaling pathways also reduces the levels of iNOS, TNF-α, and COX-2.

In another finding, an in vivo study conducted by Ichikawa and colleagues [180] had proposed epigallocatechin gallate (EGCG) to have a significant effect of reducing IL-6 expression, IKK activation, IκBα degradation, and activation of NF-κB which in turn inhibited inflammatory events that mediated by this pathway. The inhibition also reduces myocardial damage after ischemia and reperfusion in rats [181]. Furthermore, these compounds show promising effects in inhibiting IκBα degradation and the action of NF-κB in binding to DNA that leads to suppressing IL-12p40 and iNOS expression in LPS-activated macrophages [180]. In addition to EGCG, quercetin has been reported to have good anti-inflammatory activity through the same mechanism as EGCG, inhibiting IκBα phosphorylation and downmodulating the NF-κB pathway [182].

Growing evidence has been demonstrated that compounds that can inhibit JNK, p38, and IKKβ generally exhibit anti-inflammatory effects and suggest a relationship between MAPK and NFκB in inflamematory events. Flavonoid derivatives have been reported to affect the MAPK signalling pathway at various stages. A study has proven that EGCG is able to influence this pathway through the inhibition of ERK1/2, p-JNK, and p-p38 in human RA synovial fibroblasts (RASFs) [183]. Another study reported that kaempferol, luteolin, chrysin, and apigenin exert antiinflammatory activity by inhibiting MAPK signalling pathways such as JNK, p38, and ERK [181].

## Application of nanomedicine as delivery system of flavonoid derivates

Herbs have been widely used to promote human health since ancient times. Advances in phytochemistry and pharmacology have made it possible to determine the composition and bioactivity of many medicinal products. It has been proved that the effectiveness of many herbal medicines depends on the delivery of the active compounds. However, their success in clinical trials has been less impressive, partly due to the compound's poor bioavailability [184,185]. Furthermore, the efficacy of many drugs obtained from natural sources, including flavonoids in their micro/macro formulations, has also been shown to have poor bioavailability and pharmacokinetics following their oral administration, leading to their less effectivity [184,186]. Researchers have developed other drug delivery methods while avoiding potential causes of decreased bioavailability, such as first-pass metabolism and drug malabsorption in the digestive tract [187,188].

Recently, the use of nanotechnology has shown tremendous success in the field of drug delivery, including natural compound-based drugs for the treatment of inflammation. This technique formulates the natural compounds in the form of nanosized particles (1 to 100 nm) [189,190]. It has been suggested that the encapsulation of flavonoids into nanocarriers is a beneficial technique to protect drug molecules from changes in their size, shape, and surface characteristics that might be caused by environmental factors. It has been proved that this method can increase the bioavailability of flavonoids and improve the targeted and controlled release profile of the natural products, which in turn increases the drug's effectiveness [185]. Furthermore, nano-delivery systems could protect the therapeutic agents from being enzymatically metabolized, resulting in their increased stability and circulation time [191-195]. The smaller size of the nanoparticles has also been proven to increase the penetration of the compounds compared to conventional topical formulations [196]. Recently, the most common types of nanoparticles used for drug delivery are polymer nanoparticles, including solid lipid nanoparticles, liposomes, micelles, crystalline nanoparticles, and dendrimers [185,197,198].

### Solid lipid nanoparticles

Solid lipid nanoparticles (SLNs) are nanometer colloidal carriers composed of the solid particle lipid core in which active substances are trapped and stabilized by surfactants [199]. Their matrix is typically composed of solid lipids such as glycerides, sterols, fatty acids, and waxes. In particular, SLN has recently been proposed for oral and dermal administration of phenolic compounds to protect them from chemical degradation [200]. Tan *et al.* [201] demonstrated that the formulation of total flavonoid-based solid lipid nanoparticles (TF-SLN) could produce spherical particles in shape with a uniform size distribution of 104.83 nm and a zeta potential of 28.7 mV. Furthermore, this formulation was proved to have a better myocardial protection effect than flavonoids alone. Another study showed that the formulation of quercetin-loaded solid lipid nanoparticles (QSLNs) exhibited an increase in their bioavailability by 3.5-fold compared to quercetin alone in rats [12].

### Polymeric nanoparticles

Natural or synthetic biodegradable polymer nanoparticles have become prominent in the field of nanomedicine for targeted drug delivery to improve biocompatibility, bioavailability, safety, increased permeability, preferable retention time, and less toxicity [186,202]. Drugs that are conjugated to macromolecules such as synthetic polymers, natural polysaccharides, or proteins through covalent bonds provide a direct and efficient approach to modifying pharmacokinetic performance, promoting the stability of drugs, and preventing drug leakage or explosive release during transport [203].

Chitosan is the most frequently used polymer for the formulation of nano-based drugs for their delivery to their target of action. Wang *et.al.*, [204] reported that the administration of the inhaled baicalein encapsulated with chitosan-nanoparticle could mediate delivery of this drug and lead to the reduction of IL-5 level, enhancement of IL-12 and controlled inflammation-related asthma in mice. In addition, polymer nanoparticles are also effective for mediating drug delivery given topically. A study by Nan *et.al.* [205] revealed that the formulation of a topical preparation of quercetin-loaded chitosan nanoparticles led to the increase of percutaneous absorption and retention of quercetin in the skin and improve its effects against ultraviolet B radiation.

### Nanoliposomes

Nanoliposomes are phospholipid vesicles containing one or more bilayers surrounding an aqueous core. Nanoliposome-based drugs are effective for the development of transdermal drugs, topical drugs, drugs used to target hair follicles and other uses. Nanoliposomes usually consist of phospholipids with cholesterol often added to improve the stability of the liposome bilayer by filling the gaps caused by incomplete packaging. Some of the literature report that the use of nanoliposomes in natural product-based drug, especially flavonoids, is effective in delivering drugs well to the targeted sites leading to the generation of desired effects. A study by Wang *et al.* [206] explained that the formulation of nanoliposome of quercetin could induce type III-programmed cell death in C6 glioma cells. Furthermore, another study by Jin *et al.* [207] exhibited that treatment of apigenin-loaded D-alpha-tocopheryl polyethylene glycol 1000 succinate (TPGS) liposomes could deliver apigenin well and exert inhibitory effects on tumor growth in A549 cell-bearing mice.

### Nanoemulsions

Nanoemulsions are formed by combining two mechanically shear immiscible liquid phases with a surfactant having a droplet size of less than 100 nm. The formulation of natural product-based drugs with this method is able to overcome the weakness of flavonoid compounds that have problems with their bioavailability by improving their pharmacokinetic profile. A recent study by Fuior *et al.* [208] demonstrated that the formulation of flavonoid-loaded lipid nanoemulsions (FLNs) generated good characteristics (size of l200 nm, negative zeta potential, a high encapsulation efficiency >80 %, good in vitro stability, and steady release). Further, this formulation facilitated the action of the flavonoid in reducing endothelial inflammation. This finding is supported by a study conducted by Zain *et al.* [209] investigating the relationship between the delivery of nanoemulsion flavonoid-enriched oil palm and its wound-healing activity.

### Self-nano-emulsifying drug delivery system (SNEEDs)

SNEDDS is a nanoemulsion pre-concentrate in which the drug is encapsulated in an oil phase in the presence of a surfactant/cosurfactant, capable of forming very small nanoscale droplets/nanoemulsion when gently mixed with an aqueous medium. Self-emulsifying drug delivery systems are isotropic mixtures of oils, surfactants, solvents, and cosolvents/surfactants which are developed to improve the physicochemical as well as pharmacological properties of highly lipophilic drugs, including flavonoids [210-212]. It has been demonstrated that the loading of quercetin into SNEDDs could effectively enhance the hepatoprotective activity of quercetin in mice suffering from hepatotoxicity [213]. Another study performed in Wistar rats confirmed that the formulation of apigenin into SNEDDs enhanced the oral bioavailability of the compound [214].

## Concluding remarks

Flavonoids are secondary metabolites found abundantly in various plants. They exist in several subclasses, including flavanols, flavanones, flavonols, flavones, isoflavones, anthocyanins, and flavanonols. It has been demonstrated that flavonoids possess potential pharmacological activities, including their potency in mitigating inflammation. A number of studies conducted in silico, in vitro, in vivo, and clinical trials have deciphered that the anti-inflammatory activities of flavonoids are closely linked to their ability to modulate various biochemical mediators, enzymes, and signalling pathways involved in the inflammatory processes. However, these anti-inflammatory activities have been facing challenges as flavonoids have poor solubility and bioavailability. To tackle these challenges, the application of nanotechnology in developing better pharmaceutical dosage forms has brought promising results. Various nanotechnology platforms, including solid nanoparticles, polymeric nanoparticles, nanoliposomes, nanoemulsion, self-nano emulsifying drug delivery systems, have been reported to have better action in delivering flavonoids to their targeted sites. These platforms can potentially increase the solubility, bioavailability, and pharmacological activity of flavonoids and reduce the toxic effects of these compounds.

## Figures and Tables

**Figure 2.**
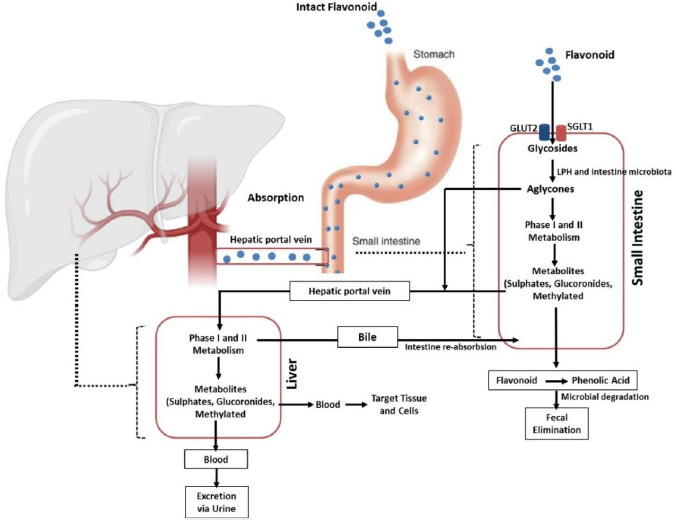
Pharmacokinetics of flavonoid.

**Table 1.** Various causes that can trigger the activation of the inflammatory cascade.

Infectious causes	Non-infectious causes
**Bacterial** Flagellin, meso-diaminopimelic acid, lipopeptides, muramyl dipeptide, lipopolysaccharides, peptidoglycans, microbial DNA, toxoid.	**Physical** Ionizing radiation, burnt, physical injury, the presence of foreign bodies, trauma, frostbite, blunt object.
**Fungi** Zymosans	**Chemical** Toxins, fatty acids, acidic environment, alcohol, and chemical irritants.
**Viruses** Single or double-stranded RNA	**Biological** Damaged or death cells
	**Allergen** Pollen, chemical compounds
	**Psychological** Excitement

**Table 2.** Subclasses of flavonoids

Subclass	Structure	Type	Ref.
Flavones	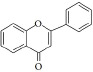	Apigenin Luteolin Acacetin Diosmetin Chrysoeriol	[41,46-50]
Flavonols	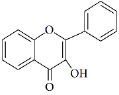	Quercetin Kaempferol Galangin Myricetin Fisetin	[51-56]
Flavanols	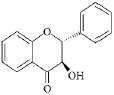	Catechin Epicatechin Gallocatechin	[57]
Flavanones	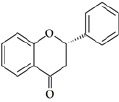	Naringenin Naringin Hesperidin Hesperetin Eriodictyol Silybin	[43,58]
Isoflavones	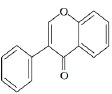	Genistein Daidzein Glycitein Formononetin Biochanin A	[59]
Anthocyanins	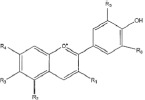	Cyanidin Malvidin Peonidin Delphinidin Pelargonidin Petunidin	[60]

**Table 3.** Anti-inflammatory activity of various flavonoids.

Class of flavonoid	Sources	Flavonoid compound	Study	Target molecules	References
Flavanol	Black and green tea, red wine, red grapes, bananas, apples, blueberries, peaches, and pears	Catechin, Epigallocatechin-3-galate, Epicatechin gallate.	In vitro and in vivo	NF-κB, IL-6, iNOS, COX-2, IL-1β, MAPK pathway, NOX, ROS, IKK, IL-8, JNK1/2, p38, JAK/STAT1 pathway, Nrf2, and PI3K/Akt	[8,89-93]
Flavanone	*Paulownia tomentosa* fruits	Tomentodiplacone O	In silico, in vitro and In vivo	COX-1, COX-2, and 5-LOX	[11]
	Citrus fruits	Hesperetin	In vitro, ex vivo and vivo	TLR4, NF-κB, TNF-α, NO, PGE_2_, iNOS, I-κB, JNK1/2, p38, MAPK and COX-2	[62,94]
	Grapefruits, sour orange, tart cherries, tomatoes, Greek oregano.	Naringenin	In vitro and in vivo	TNF-α, IL-1β, IL-6, NF-κB, Nrf2, TRPV1, TRPM3, TRPM8, caspase-3, Bad, Bax, TLR4, iNOS, COX2, NOX2, MAPK, TGF-β1, hsp70, MMP-3, IL-33, and HO-1	[95-97]
Flavanone	Citrus fruits	Naringin	In vitro, and in vivo	TNF-α, IL-1β, IL-6, IL-8, NF-κB, smad-7, TGF-β, iNOS, VEGF, pol-γ, caspase-3, smad-3, and Bax	[98-102]
	Citrus fruits	Hesperidin	In vitro and in vivo	ROS, CD14, NF- κβ, E-selectin, TNF-α, IL-1β, iNOS, IL-6, MCP-1, ICAM-1, COX-2, IFN-γ, IL-2, IL-4, IL-10, p65, Foxo1, Foxo3 and Nrf2 signaling pathway, MMP-3, MMP-9, IL-10, TIMP-1, and SOX9	[103-110]
	Citrus fruits	Eriodictyol	In vitro and in vivo	NF-κB, p38, MAPK, ERK1/2, JNK, TNF-α, IL-6, Nrf2 pathway, MIP-2, iNOS, IL-8, FOXO1, PI3K/Akt pathway, COX-2, PGE2, IκBα, p-p65, ERK1/2, JNK, NO and IL-1β	[111-114]
	Milk thistle	Silybin	In vitro and in vivo	ERK, MEK, MCP-1, IL-5, IL-10, IFN-γ, IL-17A, GM-CSF, IL-1β, TGF-β, IL-6, Ik-Bα and Raf	[115,116]
Flavonol	Berries, grapefruit, onion, olive oil, and red wine	Fisetin,	In vitro, and in vivo	MyD88 and NF-κB signaling pathways, NO, PGE2, IL-6, TNF-α, iNOS, COX-2a, IL-1β, Ser9, β-catenin, IL-2, IL-4, IFN-γ, IL-18 and IL-5	[117,118]
		Kaempferol	In silico, in vitro, in vivo, and clinical trial	MyD88 and NF-κB signaling pathways, IL-6, IL-1β, IL-18, IL-8, TNF-α, Akt, Src, Syk, IRAK1, IRAK4, Nrf2, TLR4, iNOS, IL-10, IL-12, p70, LOX, ROS, ICAM-1, E-selectin, CRP, COX1 and COX2	[51, 119-124]
		Myricetin	In vitro, and In vivo	NO, iNOS, PGE2, COX-2, TNF-α, IL-6, NF-κB, IκBα, Nrf2, STAT1, I FN-β, IL-12, IL-1β, JAK/STAT1, NOX2/p47 phox, RANKL/RANK, MAPK, MALAT1, AKT, IKK, Akt, and mTOR.	[55, 125-127]
		Quercetin	In vitro, in vivo and clinical trial	TNF-α, IL-6, IL-8, IL-1β, IL-18, IL-12, NF-κB, JNK1/2, c-Jun, ERK1/2, MCP-1, NO, iNOS and COX-2, AMPK, Sirt1, CD80, CD86, Dabs, Src, PI3K, Akt, MHC-II.	[128-133]
Flavone	*Magnolia officinalis*, fruit peels, red peppers, and tomato skins	Apigenin	In vitro, and In vivo	COX-2, NF-κB, TLR4, TGF-β, TNF-α, IL-1β, IL-6, IL-2, IL-8, NO, iNOS, AP-1 (c-Jun, c-Fos, and JunB), ERK, MCP-1, IκBα, ICAM-1, ROS, Akt, mTOR, JNK, and p38-MAPK	[134-139]
		chrysin	In vitro, and In vivo	IL-1β, IL-6, NLRP3, TGF-β, TNF-α, NF-κB	[140-142]
		luteolin	In silico in vitro, and In vivo	NF-κB, MAPK, AP- 1, SOCS3, STAT3, IL-1β, IL-6, IL-2, IL-8, IL-12, IL-10, IL-17, TNF-α, IFN-β, CCL2, CXCL2, CXCL8, and CXCL9, NLRP3, TGF-β, NF-κB, iNOS, NO, COX– 2, PGE2, GM-CSF, MCP– 1. MMP– 2 and MMP– 9.	[46]
Isoflavone	Soya bean and peanuts	Daidzin	In vitro and In vivo	TNF-α, IL6, IL-1β, IL-8, NO, iNOS, Cxcl2, PPARα/γ, p38, IκB-α, STAT1, Ccl2, and JNK	[143-146]
		genistein	In vitro and In vivo	IL-6, IL-1β, IL-8, IL-12, IL-20, IL-23, IFN-γ, TNF-α, VEGFA, CCL2, TNF-α, NF-κB, PGs, iNOS, ROS, AMPK, MAPK, JNK, TLR4, IκBα, IKK, COX-2, MCP-1, KC, ICAM-1 and VCAM-1	[147,148]
Anthocyanins	Cherry, Elsberry, blueberry, hibiscus plants, and strawberry	Cyanidin, delphinidin, malvidin	In vitro and in vivo	NF-κB, MAPK, MEK1/2-ERK1/2, JNK, IL-1β, IL-6, TNF-α, COX-2, PGE2, iNOS, NO, MCP-1, ICAM-1, VCAM-1, and CINC-1.	[149-154]
Flavanonols	Milk thistle	Silibinin	In vitro, in vivo, and clinical trial	NF-κB, MAPK, TNF-α, IL-6, IL-8, IL-1β, IL-10, MMP-9, COX-2, PGE2, PGF2α, Th17, SIRT1, cyclin D1, Bcl-2, iNOS, NO, VEGF, MMP-1, MMP-3, MMP-13, PI3K/Akt	[88,155-159]
